# Annual Meeting

**Published:** 1921-07-09

**Authors:** 


### THE BRITISH HOSPITALS ASSOCIATION.

### Annual Meeting.

The annual meeting of the British Hospitals Association was held on Friday, July 1, at the Manchester Royal Infirmary. The President (the Hon. Sir Arthur Stanley, G.B.E.) was supported by Sir William Cobbett (Vice-President) and Mr. H. Wade Deacon, C.B.E., Chairman of the Council.

Mr. Wade Deacon moved the adoption of the annual report, and expressed satisfaction at the formation of sixteen Regional Committees, nine of which have already nominated members to serve on the Council. The election of Mr. Conrad W. Thies to the office of Vice-President marked a special expression of gratitude from the members for all he had done to further the work and usefulness of the Association since its reconstitution in January 1910. Thanks were due and were cordially expressed to the President and the honorary officers of the Association—the Hon. Secretary, Mr. J. Courtney Buchanan, C.B.E., deserving a special measure of gratitude for his untiring efforts to promote the interests of the Association. The President and honorary officers were re-elected, together with the existing members of the Council and nine new Council members.

A resolution was passed welcoming the Voluntary Hospitals Committee's report, and expressing appreciation of the care and consideration given to the various aspects of hospital difficulties and problems laid before Lord Cave's Committee of Inquiry. Sir Arthur Stanley said he had been called as a witness at the inquiry, and had watched its proceedings from beginning to end. He was able to say that, as a result of the Committee's investigation, the voluntary system came out more strongly than ever. Government Departments, and everyone concerned, had put it definitely on record that every effort ought to be made to preserve the voluntary system for the hospitals, so that they should not be put on the rates or in the hands of the State. "That, from our point of view," said Sir Arthur, "is most satisfactory."

### Approved Societies' Action.

The question of how far, and in what manner, the Approved Societies should contribute towards the upkeep of the hospitals of the country, was discussed. Sir Arthur Stanley referred to Dr. Gordon Dill's scheme, which, he said, was a definite scheme to be contributed to by people who could afford it, not for the treatment they might receive in a hospital, but for the treatment they hoped not to receive. "The payment of fire insurance is possible," he went on, "not because people's houses are burnt down, but because their houses are not burnt down, and if we can get a scheme of insurance by which a large number of people who don't require hospital treatment are insured against it, then I think we shall get a very satisfactory result for the hospitals." It was difficult to say what proportion of the population had treatment in the hospitals every year. The figure given originally was 2 per cent., but that was probably too low, and the Association had put it at 5 per cent. If the other 95 per cent. were insured in the manner indicated, the hospitals should get a good income. It had been suggested, Sir Arthur said, that the Approved Societies should contribute a guinea a week for each member treated in hospital. That, however, fell far below the cost. He thought it was clear that the hospitals would not get any of the surplus accumulated by the Approved Societies in the past—which the hospitals had helped them to accumulate—but there was no reason why they should not try to get some of the surplus which the societies would amass in the future. In further discussion, the opinion was expressed that a system of regular contributions from the Approved Societies might be inimical to the hospitals as casting a doubt on their voluntary character. This, however, did not seem to be the general view.

In the end, the meeting authorised the Council of the Association to continue its negotiations with the Approved Societies on the question, and to obtain from them the best possible terms. The hope was expressed for a block grant, if such could be arranged, in preference to a *per capita* payment. It was further resolved that hospitals should have a free hand to deal with the smaller local associations, the Executive Committee dealing with the Approved Societies collectively. A resolution was also passed that the General Nursing Council should be requested not to put their syllabus definitely in operation until the opinion of the hospitals has been heard.

Amongst those present were the following representatives of thirty-two hospitals, twenty-two of which are situated in the north-western region: C. H. Ingram and E. Hemingway (Dewsbury), H. L. Boot (Warrington), J. H. Shaw (Southport), Jas. E. Ludham and F. Oliver (Ashton-under-Lyne), Capt. Currie and J. R. Mitchell (Chester Royal Infirmary), E. J. Pearce (Stockport), Dr. Fawsett and C. D. Drake (Oldham), F. Burnicle and S. C. Fryers (Sunderland Royal Infirmary), J. Gibson (Preston), H. Wade Deacon, H. Harrison, and W. Rutter (Liverpool Royal Infirmary), G. Ruddle and L. Sumner (Salford), J. K. Milward (Newport), T. Wilson (Manchester Dental Hospital), Rev. J. E. Samuel and N. A. Smith (Blackburn), H. H. Bedford (Sheffield Royal Infirmary), J. A. Cowley (Northwich), Canon Hill (Bury Infirmary), R. Ratcliffe and E. Reid (St. Mary's, Manchester), J. B. Sleight and J. J. Barron (Bradford Royal Infirmary), B. Frankenburg (Greengate Dispensary, Manchester), R. Barrow Sicree and Mrs. Quas Cohen (Manchester Jewish Hospital), S. Brunt (Wigan), Sir William Cobbett and F. G. Hazell (Manchester Royal Infirmary), C. Risbee (Northampton General Hospital), W. H. Harper (Wolverhampton), Rev. G. B. Cronshaw (Oxford), H. J. Dafforne (Ancoats Hospital), A. Naldrett (Liverpool Royal Southern), H. E. Vipan (Victoria Central Hospital, Wallasey), E. W. Osborne (Liverpool Stanley Hospital), and J. Courtney Buchanan (Cancer Hospital, London), Hon. Secretary.

